# Phase-Controlled Synthesis of Ru Supported on Carbon Nitride and the Application in Photocatalytic H_2_ Evolution

**DOI:** 10.3390/ma18061259

**Published:** 2025-03-13

**Authors:** Xiaohu Sun, Xiangyang Cao, Ganghua Zhou, Tiaolong Lv, Jian Xu, Yubo Zhou, Zhigang Wang, Jianjian Yi

**Affiliations:** 1 College of Environmental Science and Engineering, Yangzhou University, Yangzhou 225127, China; mz120211186@stu.yzu.edu.cn (X.S.);; 2 Ningbo Solartron Technology Co., Ltd., Ningbo 315034, China; 3 School of Material Science and Engineering, Jiangsu University, Zhenjiang 212013, China

**Keywords:** phase engineering, ruthenium, photocatalysis, hydrogen evolution, co-catalyst

## Abstract

This work aims to explore the influence of crystal phase engineering on the photocatalytic hydrogen evolution activity of Ru/C_3_N_4_ systems. By precisely tuning the combination of Ru precursors and reducing solvents, we successfully synthesized Ru co-catalysts with distinct crystal phases (hcp and fcc) and integrated them with C_3_N_4_. The photocatalytic hydrogen evolution experiments demonstrated that hcp-Ru/C_3_N_4_ achieved a significantly higher hydrogen evolution rate (24.23 μmol h^−1^) compared to fcc-Ru/C_3_N_4_ (7.44 μmol h^−1^), with activity reaching approximately 42% of Pt/C_3_N_4_ under the same conditions. Photocurrent and electrochemical impedance spectroscopy analyses revealed that hcp-Ru/C_3_N_4_ exhibited superior charge separation and transfer efficiency. Moreover, Gibbs free energy calculations indicated that the hydrogen adsorption energy of hcp-Ru (ΔG_H*_ = −0.14 eV) was closer to optimal compared to fcc-Ru (−0.32 eV), enhancing the hydrogen generation process. These findings highlight that crystal-phase engineering plays a critical role in tuning the electronic structure and catalytic properties of Ru-based systems, offering insights for the design of highly efficient noble metal catalysts for photocatalysis.

## 1. Introduction

The development of more efficient nanomaterial catalysts is crucial in fields such as environmental protection, food processing, petrochemicals and energy utilization. Catalyst performance can be enhanced by controlling the nanostructures through strategies involving size [1], shape [2], alloys [3], crystal phase [4], and defects [5]. Phase engineering of nanomaterials (PEN), as an effective strategy for the rational design and precise synthesis of nanomaterials with controllable phases, has gained significant attention in recent years [6,7,8,9]. Phase engineering modulates performance, functionality, and applications by altering the catalyst’s surface electronic and geometric structures through adjustments in reaction kinetics and surface energy [10,11,12]. For instance, bulk Nickel (Ni) typically shows the face-centered cubic (fcc) phase, but non-conventional hexagonal close-packed (hcp) Ni nanoparticles can be synthesized via chemical methods or one-pot procedures [13,14]. Experimental results indicate that hcp Ni exhibits superior catalytic performance compared to fcc Ni across various reactions. Additionally, studies have explored the impact of crystallinity on catalytic performance, revealing that the amorphous 1T phase of MoSe_2_ rich in unsaturated coordination sites, facilitates proton coupling to form hydrogen [15]. This provides new insights into phase engineering for fine-tuning growth modes. Nevertheless, the practical application of phase engineering is still in its early stages, and there is an urgent need for the development of environmentally friendly and versatile synthetic methods for phase regulation. Furthermore, the influence mechanisms of template, reduction kinetics, and capping agents on atomic stacking require further investigation.

Photocatalytic hydrogen production is considered a green, clean, safe, and low-cost renewable energy technology. Due to their high electron capture efficiency, excellent chemical stability, and abundant surface active sites, noble metals play an essential role in the preparation of photocatalysts for hydrogen production [16,17,18,19]. Among them, platinum (Pt), with its optimal Fermi level and zero-approaching hydrogen adsorption Gibbs free energy, is one of the most promising co-catalysts for photocatalytic hydrogen production [20]. However, due to its high cost, large-scale use remains impractical. To reduce the dependence on expensive Pt catalysts for photocatalytic water splitting, researchers have explored various types of catalysts and optimized their nanostructures to develop high-activity, low-cost co-catalysts. Ruthenium (Ru) has attracted significant attention in the field of photocatalysis due to its lower cost and the ability to achieve activity comparable to Pt under certain optimized conditions [21,22,23]. Zhu et al. optimized the asymmetric electronic properties of Ru to lower the energy barrier for Ru^n+^ in water splitting, and the resulting Ru/NC catalyst exhibited high alkaline hydrogen evolution reaction (HER) activity (21.9 mV @ 10 mA cm^−2^, 29.03 mV dec^−1^) [24]. Dong et al. discovered that the incorporation of copper (Cu) in the alloy altered the reaction pathway for Ru in photocatalysis, achieving a 96% selectivity for CO_2_ to CH_4_ [25]. Despite numerous successful cases of Ru in various catalytic reactions, studies on the synthesis and optimal crystal phase of Ru co-catalysts for hydrogen production are scarce, and the mechanisms underlying the impact of Ru crystal phase on photocatalytic performance remain largely unexplored. Therefore, it is essential to investigate the feasibility of phase control in Ru for enhancing hydrogen production.

In this study, we employ a rational and controllable chemical reduction method to prepare hcp and fcc Ru nanoparticles and successfully load them onto C_3_N_4_ ultrathin nanosheets (hcp-Ru/C_3_N_4_, fcc-Ru/C_3_N_4_) to demonstrate the feasibility of improving the photocatalytic hydrogen production performance of Ru-based co-catalysts using phase engineering. Experimental results show that the Ru-based co-catalysts exhibit distinct phase-dependent behavior. hcp-Ru/C_3_N_4_ displays more efficient photocatalytic hydrogen production activity (24.23 µmol h^−1^) and stability, significantly outperforming fcc-Ru/C_3_N_4_ (7.44 µmol h^−1^). This superior performance is attributed to its higher charge separation efficiency and lower Gibbs free energy, as supported by detailed physicochemical characterization and theoretical calculations. This study highlights the vast potential and versatility of Ru phase-controlled synthesis for improving photocatalytic hydrogen production.

## 2. Materials and Methods

### 2.1. Materials

Ruthenium acetylacetonate (Ru(acac)_3_, C_15_H_21_O_6_Ru, ≥97%), triethylene glycol (C_6_H_14_O_4,_ >99%), triethanolamine (TEOA, C_6_H_15_NO_3_, >99%), polyvinylpyrrolidone ((C_6_H_9_NO)_n_, K30, MW ≈ 40,000) were purchased from Shanghai Aladdin Biochemical Technology (Shanghai, China). Melamine (C_3_H_6_N_6_, ≥99.0%), ruthenium (III) chloride hydrate (RuCl_3_·xH_2_O), ethanol absolute (C_2_H_6_O, ≥99.7%), ethylene glycol (C_2_H_6_O_2_, ≥99.5%), potassium bromide (KBr, ≥99.0%) and sodium sulfate (Na_2_SO_4_, ≥99.0%) were purchased from Sinopharm Chemical Reagent Co., Ltd. (Shanghai, China).

### 2.2. Methods

#### 2.2.1. Synthesis of C_3_N_4_ Nanosheets

An amount of 2 g of melamine was weighed and added to a covered ceramic crucible. The crucible was then placed in a muffle furnace and heated to 550 °C at a rate of 2 °C/min, where it was held for 4 h. After the reaction, the resulting yellow block was obtained as the bulk material. The bulk material was ground into a powder, and 800 mg of the powder was spread evenly in a partially open ceramic boat. The boat was then placed in the muffle furnace and heated to 550 °C at a rate of 10 °C/min, where it was held for 40 min. The resulting white powder was the desired C_3_N_4_ for the experiment.

#### 2.2.2. Synthesis of C_3_N_4_-Ru Samples

In the synthesis of hcp-Ru/C_3_N_4_, 10 mL of an ethylene glycol (EG) solution containing 50 mg of C_3_N_4_ nanosheets and 0.5 mmol of polyvinylpyrrolidone (PVP) was sonicated in a 25 mL round-bottom flask for 30 min. Then, 0.025 mmol of RuCl_3_·xH_2_O was added to the flask and sonicated until well dispersed. The flask containing the reaction mixture was then transferred to an oil bath and heated to 200 °C for 3 h. After the reaction was complete, the sample was washed with ethanol and deionized water, and the product was separated by centrifugation, followed by freeze-drying to obtain hcp-Ru/C_3_N_4_.

In the synthesis of fcc-Ru/C_3_N_4_, 10 mL of a triethylene glycol (TEG) solution containing 50 mg of C_3_N_4_ nanosheets and 0.5 mmol of PVP was sonicated in a 25 mL round-bottom flask for 30 min. Then, 0.025 mmol of Ru(acac)_3_ was added to the flask and sonicated until well dispersed. The flask containing the reaction mixture was then transferred to an oil bath and heated to 200 °C for 3 h. After the reaction was complete, the sample was washed with ethanol and deionized water, and the product was separated by centrifugation, followed by freeze-drying to obtain fcc-Ru/C_3_N_4_.

### 2.3. Photocatalytic Hydrogen Activity Measurement

The photocatalytic hydrogen evolution reaction was carried out in an online reaction detection system (CEL-SPH2N, China Education Au-Light, Beijing, China). The reaction solution consisted of 50 mL of TEOA/H_2_O (10% vol TEOA). The photocatalyst used in the measurement was 10 mg. The reaction was irradiated with a 300 W xenon lamp (CEL-HX F300/CEL-HX UV300, China Education Au-Light, Beijing, China) without any filters. Prior to irradiation, the system was evacuated to a vacuum state, and the reaction temperature was maintained at 15 °C using a circulating cooling water system. The hydrogen production rate was analyzed by gas chromatography (GC 7920) equipped with a TCD detector and a 5Å molecular sieve column (all components purchased from China Education Au-light, Beijing, China), using argon as the carrier gas.

The external quantum efficiency (*EQE*) values were determined using the following equation:

$$EQE=\frac{the\;number\;of\;reacted\;electrons}{the\;number\;of\;incident\;photons}\times 100\%$$

A more detailed calculation process can be found in the Supplementary Materials, including the information of the area of light spot, light intensities per unit area, and wavelength-dependent hydrogen evolution rates per unit area.

## 3. Results

**Figure 1:**
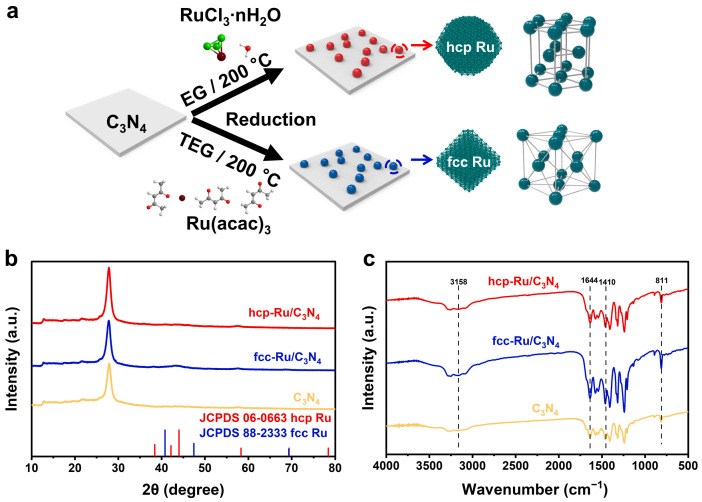
(**a**) Schematic illustrating of the synthesis process of hcp-Ru/C_3_N_4_ and fcc-Ru/C_3_N_4_. (**b**) XRD patterns and (**c**) FT-IR spectra.

The premise for studying the impact of crystal phase structure on photocatalytic reactions is the development of methods for controlling the crystal phase synthesis of Ru. We first prepared ultrathin C_3_N_4_ nanosheets as a support using a top-down thermal exfoliation method. Their coiled two-dimensional structure and large surface area are expected to provide an ideal environment for Ru loading. Different precursors and reducing agents can synergistically regulate the reduction kinetics rate and play an important role in selectively forming hcp and fcc phases of Ru [26,27]. The strong reducing nature of EG and the more easily dissociable chloride ligands in RuCl_3_ molecules favor the formation of hcp Ru seeds. In contrast, TEG, with its weaker reducing ability, and the more stable acetylacetonate ligands in Ru(acac)_3_ act to uniformly release Ru atoms, promoting the formation of the fcc structure. Therefore, in the synthesis of hcp-Ru/C_3_N_4_, RuCl_3_·xH_2_O was used as the Ru source, EG as the reducing agent and solvent, and PVP as the stabilizer, to chemically reduce and load hcp phase Ru nanoparticles onto the C_3_N_4_ nanosheets. The synthesis of fcc-Ru/C_3_N_4_ followed a similar method, with the only difference being the use of Ru(acac)_3_ as the Ru source and TEG as the reducing agent and solvent (Figure 1a).

To understand the basic chemical structure of the prepared catalysts, we studied their crystal structure using X-ray diffraction (XRD). The XRD patterns of the prepared hcp-Ru/C_3_N_4_ and fcc-Ru/C_3_N_4_ exhibited features similar to those of C_3_N_4_ (Figure 1b). Two characteristic diffraction peaks of C_3_N_4_ were detected at 2θ angles of 12.8° and 27.8°, corresponding to the stacking units of the in-planar repeating tri-*s*-triazine unit and the conjugated aromatic segments, respectively. These peaks correspond to the (100) and (002) crystal planes [28]. No characteristic peaks of Ru were detected in the XRD patterns of hcp-Ru/C_3_N_4_ and fcc-Ru/C_3_N_4_ due to the low content and small particle size, which were undetectable [29]. This was later confirmed through transmission electron microscope (TEM, Tecnai G2 F30 S-TWIN, purchased from FEI COMPANY, Hillsboro, OR, USA), which calculated the average particle size of Ru. Fourier transform infrared (FT-IR, Nicolet iS5, purchased from Thermo Fisher Scientific, Waltham, MA, USA) spectra were used to identify the surface functional groups of hcp-Ru/C_3_N_4_, fcc-Ru/C_3_N_4_, and C_3_N_4_ (Figure 1c). The characteristic peaks of C_3_N_4_ were clearly observed in all the catalysts. The absorption peak at 811 cm^−1^ corresponds to the characteristic vibration of the triazine units. The absorption peaks at 1410 and 1644 cm^−1^ are attributed to the stretching vibrations of C-N heterocyclic bonds, and the absorption peak at 3158 cm^−1^ corresponds to the stretching vibration of the O-H bond [30]. These results demonstrate that the introduction of Ru does not alter the original fundamental functional group structure of C_3_N_4_.

**Figure 2:**
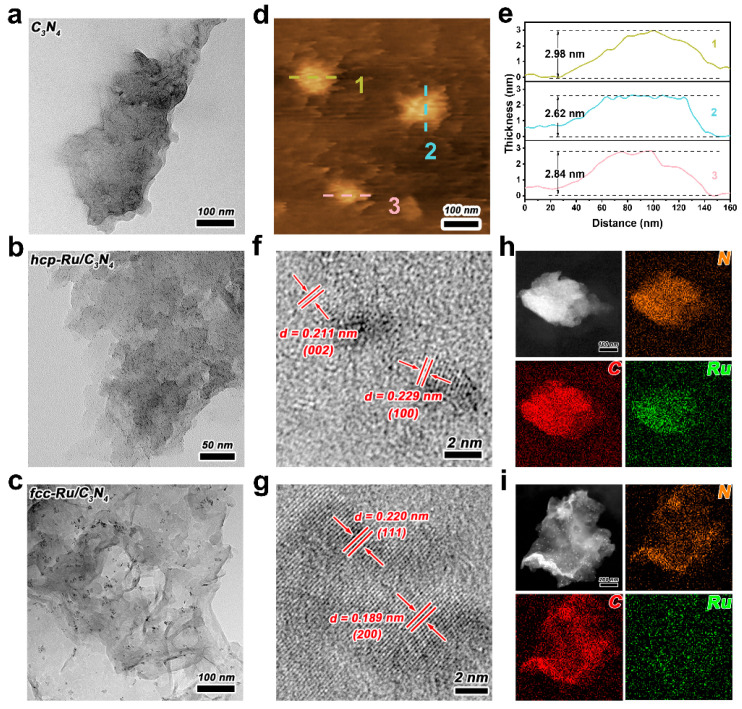
(**a**–**c**) TEM images of C_3_N_4_, hcp-Ru/C_3_N_4_ and fcc-Ru/C_3_N_4_. (**f**,**g**) HRTEM images and (**h**,**i**) STEM and elemental mapping images of hcp-Ru/C_3_N_4_ and fcc-Ru/C_3_N_4_. (**d**) AFM image of C_3_N_4_. (**e**) Thickness profile in (**d**).

After determining the basic chemical structure of the prepared catalysts, we further investigated their microstructure and chemical composition through a series of electron microscopy characterizations. Scanning electron microscope (SEM, S-4800II, purchased from HITACHI, Tokyo, Japan) results show that the prepared C_3_N_4_ exhibits irregular porous coiled folds (Figure S1a,b). Atomic force microscope (AFM, SPM-9700HT, purchased from Shimadzu, Tokyo, Japan) observed that its thickness is approximately 2.8 nm (Figure 2d,e), providing further evidence of its typical ultrathin two-dimensional nanosheet structure. The larger lateral dimensions and Brunauer–Emmett–Teller (BET, ASAP2460, purchased from Micromeritics Instruments Corporation, Atlanta, GA, USA) surface area (Figure S7b–d) of C_3_N_4_ facilitate Ru loading, while the ultrathin thickness effectively shortens the charge migration path from the bulk to the surface, thereby reducing electron–hole recombination [31,32]. After loading with metallic Ru nanoparticles, the morphology of C_3_N_4_ did not show significant changes, and both hcp-Ru/C_3_N_4_ and fcc-Ru/C_3_N_4_ composite photocatalysts still maintained an irregular nanosheet morphology (Figure S1c–f). TEM results of hcp-Ru/C_3_N_4_ and fcc-Ru/C_3_N_4_ show that the Ru nanoparticles are uniformly dispersed on the flat surface of C_3_N_4_, with no apparent aggregation (Figure 2a–c), which can be attributed to the appropriate amount of reducing agent and PVP that control the uniform growth of metal Ru nanoparticles, preventing aggregation on C_3_N_4_ [33]. In addition, we have supplemented TEM-based size distribution analyses for both hcp-Ru (1.03 nm) and fcc-Ru (1.54 nm) samples (Figure S2a,b). While a minor size difference exists between the two phases (~0.5 nm), we propose that this subtle variation has negligible impacts on catalytic performance trends [34,35].

To reveal the fine-phase structure of the Ru loaded on the catalyst, we performed high-resolution transmission electron microscope (HRTEM, Tecnai G2 F30 S-TWIN, purchased from FEI COMPANY, Hillsboro, OR, USA) characterization. The lattice spacings of 0.211 and 0.229 nm in Figure 2f correspond to the (002) and (100) planes of hcp Ru, while the lattice spacings of 0.189 and 0.220 nm in Figure 2g correspond to the (200) and (111) planes of fcc Ru. Additional HRTEM images are presented in Figure S3 to further clarify the phase purity of Ru nanoparticles in the catalysts. This indicates that the method used in this work successfully controls the crystal phase of Ru. Elemental mapping images further confirmed the uniform distribution of C, N, and Ru elements (Figure 2h,i), providing additional evidence that Ru nanoparticles of both crystal phases are uniformly loaded on C_3_N_4_. Furthermore, the actual Ru loading amount on hcp-Ru/C_3_N_4_ and fcc-Ru/C_3_N_4_ was determined to be 3.53 wt% and 3.64 wt% by ICP-MS (Elan DRC-e, purchased from PerkinElmer, Waltham, MA, USA) (Table S1).

**Figure 3:**
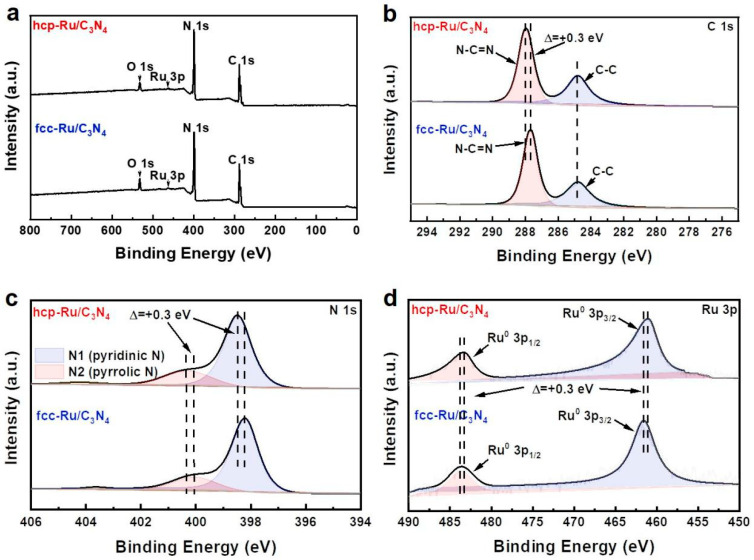
(**a**) XPS survey spectra and XPS spectra of (**b**) C 1s, (**c**) N 1s and (**d**) Ru 3p of hcp-Ru/C_3_N_4_ and fcc-Ru/C_3_N_4_.

X-ray photoelectron spectroscopy (XPS, ESCALAB 250Xi, purchased from Thermo Fisher Scientific, Waltham, MA, USA) was used to characterize the element valence states and surface composition of the catalysts. Full XPS spectra of hcp-Ru/C_3_N_4_ and fcc-Ru/C_3_N_4_ clearly showed signals corresponding to C, N, and Ru, further confirming the successful synthesis of the catalysts (Figure 3a). The C 1s spectrum of hcp-Ru/C_3_N_4_ (Figure 3b) can be deconvoluted into two peaks at 284.8 eV and 287.9 eV, corresponding to graphitic carbon (C-C) and sp^2^-bonded aromatic structure (N-C=N), respectively. In the N 1s spectrum (Figure 3c), binding energy of 398.5 eV and 400.3 eV correspond to pyridinic N (N1) and pyrrolic N (N2) [36]. The Ru 3p spectrum (Figure 3d) reveals the Ru 3p_3/2_ and Ru 3p_1/2_ spin–orbit peaks, with fitting peaks at 461.2 eV and 483.5 eV assigned to metallic Ru (Ru^0^) [37]. In contrast, the C 1s and N 1s spectra of fcc-Ru/C_3_N_4_ show a more negative binding energy shift (~0.3 eV), and the signal peaks in the Ru 3p spectrum are shifted positively by 0.3 eV. This suggests that C_3_N_4_ interacts more strongly with hcp Ru, with more electrons transferred to hcp Ru [38]. Despite the differences in the crystal phase structure of Ru loaded on the surfaces of hcp-Ru/C_3_N_4_ and fcc-Ru/C_3_N_4_, their similar XPS spectra indicate that both catalysts possess similar chemical valence states.

**Figure 4:**
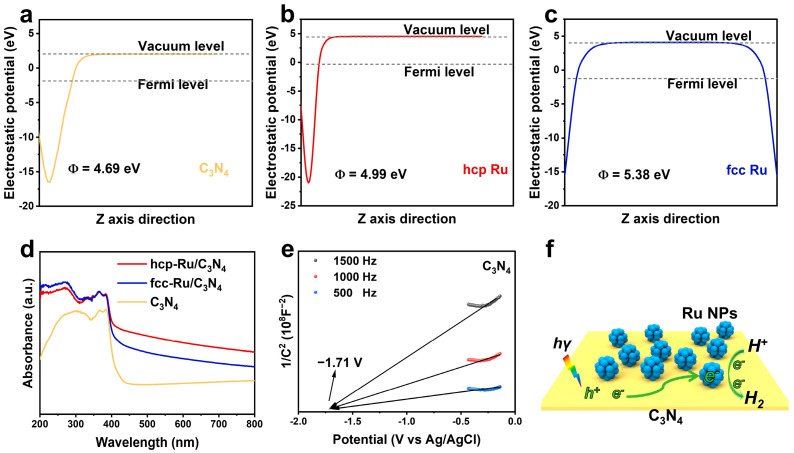
(**a**–**c**) Average potential profiles along the z-axis direction and calculated work function values of C_3_N_4_, hcp-Ru/C_3_N_4_ and fcc-Ru/C_3_N_4_. (**d**) UV-Vis diffuse reflection spectra of hcp-Ru/C_3_N_4_, fcc-Ru/C_3_N_4_ and C_3_N_4_. (**e**) Mott–Schottky plots of C_3_N_4_. (**f**) Schematic diagram of photocatalytic H_2_ evolution process on the surface of hcp-Ru/C_3_N_4_ and fcc-Ru/C_3_N_4_.

After confirming the fine chemical structure of the prepared catalysts, we further discussed the rationality of constructing the Ru/C_3_N_4_ photocatalytic system using density functional theory (DFT). From a thermodynamic perspective, the direction of charge transfer is from the component with a higher Fermi level (i.e., lower work function) to the component with a lower Fermi level (i.e., higher work function) [39]. As shown in Figure 4a–c, we first calculated the theoretical work functions of C_3_N_4_, hcp-Ru, and fcc-Ru using DFT, which were found to be 4.69, 4.99, and 5.38 eV, respectively. The data indicate that after loading Ru onto C_3_N_4_, the photogenerated electrons produced upon light excitation can thermodynamically transfer to Ru, thereby enhancing charge separation efficiency and the electron concentration on the catalyst surface, providing theoretical support for Ru’s role as a co-catalyst.

Next, we assessed the light absorption ability of the catalysts using UV-Vis diffuse reflection spectra (DRS) (Figure 4d). It was found that C_3_N_4_ has a typical absorption edge of around 450 nm. After loading Ru, the optical absorption properties of the material were improved, and the visible light absorption range increased. This is mainly due to the synthesized catalyst being black in color. However, it should be noted that this enhancement in absorption is not significant, as the increased light absorption comes from Ru, which cannot be directly excited by light. The light absorption and charge generation capacity are solely determined by C_3_N_4_. Therefore, it can be concluded that both hcp-Ru/C_3_N_4_ and fcc-Ru/C_3_N_4_ exhibit similar charge generation behaviors.

Based on the theoretical analysis, we further determined the electric potential of C_3_N_4_ and speculated on its charge transfer pathway through Tauc curves and Mott–Schottky (M–S) plots. From the Tauc curve data, the bandgap of C_3_N_4_ was calculated to be 3.03 eV (Figure S4a). The flat band (FB) potential of C_3_N_4_ was determined to be −1.71 V vs. Ag/AgCl, pH = 7, using the M–S curves (Figure 4e) at 500, 1000, and 1500 Hz. Assuming that for n-type semiconductors, the gap between the flat band potential and conduction band (CB) potential can be neglected [40]. The conduction band potential of C_3_N_4_ was converted to −1.1 V vs. NHE, pH = 0 [41]. Thus, its valence band (VB) potential was determined to be 1.93 V vs. NHE, pH = 0, and the band structure was obtained (Figure S4b). Therefore, we can speculate on the charge transfer pathway in the Ru-C_3_N_4_ catalyst during photocatalytic hydrogen evolution (Figure 4f). Upon light irradiation, C_3_N_4_ generates electron–hole pairs. The photogenerated electrons migrate through the interface to the Ru surface. Due to the rich hydrogen evolution active sites at the edge and basal plane of Ru, the electrons gathered on its surface react with adsorbed protons to produce H_2_. Meanwhile, the holes generated in C_3_N_4_ are consumed by the sacrificial agent, preventing the recombination of electron–hole pairs before charge separation and consumption.

**Figure 5:**
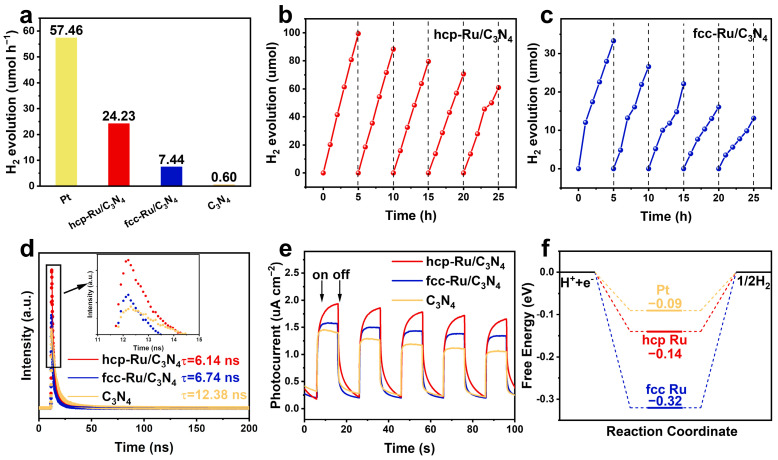
(**a**) Photocatalytic hydrogen evolution rates for C_3_N_4_-based hybrids loaded with phase-engineered Ru. (**b**) Cycle experiment of hcp-Ru/C_3_N_4_. (**c**) Cycle experiment of fcc-Ru/C_3_N_4_. (**d**) transient-state spectra of the catalysts. (**e**) Photocurrent vs. time (I-t) curves of the catalysts. (**f**) Calculated Gibbs free energy diagrams for hydrogen evolution at hcp Ru and fcc Ru surfaces.

In actual photocatalytic experiments, the hydrogen evolution activity of both Ru/C_3_N_4_ catalysts (with a theoretical loading of 5 wt%) was significantly improved compared to the pure C_3_N_4_. The average hydrogen evolution rates are shown in Figure 5a. Among them, hcp-Ru/C_3_N_4_ (24.23 μmol h^−1^) exhibited much higher catalytic activity than fcc-Ru/C_3_N_4_ (7.44 μmol h^−1^), indicating that the crystal structure of Ru significantly affects the photocatalytic hydrogen production performance of Ru/C_3_N_4_. To better understand the hydrogen production performance of hcp-Ru/C_3_N_4_, it is essential to evaluate its external quantum efficiency (*EQE*). As shown in Table S2, the *EQE* value of hcp-Ru/C_3_N_4_ is measured to be 5.28% at 420 nm. Clearly, compared to the catalysts listed in Table S3, hcp-Ru/C_3_N_4_ demonstrates superior performance in both hydrogen evolution rate and quantum efficiency, further highlighting its exceptional photocatalytic activity. We also tested the hydrogen evolution activity of Pt/C_3_N_4_ under the same conditions, which reached 57.46 μmol h^−1^. This result indicates that the performance of the synthesized Ru co-catalyst is approximately 42% of that of Pt, showing a distinct advantage over most non-Pt co-catalysts. Additionally, we investigated the stability of hcp-Ru/C_3_N_4_ and fcc-Ru/C_3_N_4_ by conducting five photocatalytic reactions (25 h), as shown in Figure 5b,c. The results revealed that hcp-Ru/C_3_N_4_ demonstrated superior stability in hydrogen production compared to fcc-Ru/C_3_N_4_. Moreover, after the photocatalytic reaction, we performed characterizations on hcp-Ru/C_3_N_4_, which showed good chemical and structural stability (Figure S5). The slight decline in activity of hcp-Ru/C_3_N_4_ during the reaction could be attributed to the consumption of the sacrificial agent over prolonged reaction times.

To further explore the reasons for the significant difference in activity between the two Ru crystal phase catalysts, we continued to investigate their photoelectrochemical properties. We combined Linear sweep voltammetry (LSV) curves, photoelectrochemical measurements (photocurrent and electrochemical impedance spectroscopy (EIS)), steady-state fluorescence (PL), and transient-state fluorescence (FL) to further evaluate the charge dynamics of the two catalysts. As observed from the LSV curves (Figure S6b), the photocurrent density of hcp-Ru/C_3_N_4_ is higher than that of fcc-Ru/C_3_N_4_, and both are greater than that of C_3_N_4_. This indicates that Ru can significantly reduce the surface energy barrier of C_3_N_4_, enhances the consumption rate of photogenerated holes, and increases the density of free photogenerated electrons required for hydrogen evolution at lower potentials. The electrochemical hydrogen evolution activity is consistent with the photocatalytic performance, further demonstrating that hcp-Ru exhibits superior water reduction kinetics. As shown in Figure 5e, compared to C_3_N_4_, the introduction of Ru effectively enhanced the photocurrent response under light illumination, indicating that Ru loading promotes charge transfer in C_3_N_4_. hcp-Ru exhibited stronger photocurrent intensity than fcc-Ru, with the photocurrent response intensity order being: hcp-Ru/C_3_N_4_ > fcc-Ru/C_3_N_4_ > C_3_N_4_. The EIS results are consistent with the photocurrent results (Figure S6a), indicating that electrons in hcp-Ru/C_3_N_4_ exhibit more efficient transfer. Additionally, both materials showed a significant decrease in charge lifetime (Figure 5d) and a marked reduction in FL intensity (Figure S7a), further evidencing the enhanced charge separation efficiency [42,43]. Notably, although theoretical calculations of the Fermi level (Figure 4a–c) and charge difference distribution (Figure S6c) indicate that photogenerated electrons can effectively migrate from the conduction band of C_3_N_4_ to Ru nanoparticles, the electron density at the fcc-Ru/C_3_N_4_ interface is higher than that of hcp-Ru, which contradicts the actual photoelectric response and EIS results. We speculate that fcc-Ru can capture more electrons, but the water reduction kinetics on the surface of fcc-Ru is relatively poor, resulting in fewer electrons participating in surface hydrogen evolution, leading to a higher internal electron–hole recombination rate; thus, a reduced number of effective electrons transferred to the electrode. The comparison of Gibbs free energy results further supports our hypothesis.

The high photogenerated charge separation efficiency could be one of the reasons for the superior photocatalytic hydrogen evolution activity of hcp-Ru/C_3_N_4_. However, the surface molecular conversion barrier should also be considered an important factor [44]. It is evident that hcp-Ru possesses more active surface catalytic sites, corresponding to lower reaction energy barriers. Specifically, the rate-determining step for hydrogen evolution on the Ru surface is the formation of H* species. From the Gibbs free energy of this step, hcp-Ru (ΔG_H*_ = −0.14 eV) is significantly lower than fcc-Ru (ΔG_H*_ = −0.32 eV), and even approaches the efficient catalytic behavior of Pt (Figure 5f), making it easier to undergo reduction reactions at the CB edge to generate H_2_. This indicates that, compared to fcc-Ru/C_3_N_4_, hcp-Ru/C_3_N_4_ not only exhibits higher charge separation efficiency but also demonstrates better surface molecular conversion efficiency. Based on these favorable physicochemical properties induced by phase engineering, hcp-Ru/C_3_N_4_ shows higher activity and better stability in photocatalytic hydrogen evolution reactions.

## 4. Conclusions

This study highlights the significant impact of crystal phase engineering on the photocatalytic performance of Ru/C_3_N_4_ systems for hydrogen evolution. By manipulating the choice of Ru precursors and reducing solvents, we achieved precise control over the synthesis of hcp- and fcc-phase Ru catalysts. Experimental results demonstrated that the hcp-Ru/C_3_N_4_ composite displayed markedly superior hydrogen evolution activity and stability compared to its fcc counterpart, benefiting from enhanced charge separation and transfer capabilities. Thermodynamic analysis further revealed that the surface catalytic properties of hcp-Ru, characterized by an optimized hydrogen adsorption energy, play a pivotal role in improving its photocatalytic efficiency. This work provides valuable insights into the rational design of photocatalysts via crystal phase engineering, emphasizing the synergistic effects of electronic structure and surface reactivity for effective hydrogen production.

## Data Availability

The original contributions presented in this study are included in the article/Supplementary Materials. Further inquiries can be directed to the corresponding author.

## Supplementary Materials

The following supporting information can be downloaded at: https://www.mdpi.com/article/10.3390/ma18061259/s1, Figure S1: images of C_3_N_4_, hcp-Ru/C_3_N_4_ and fcc-Ru/C_3_N_4_; Figure S2: (a,b) Size distribution histograms of Ru nanocrystals on the C_3_N_4_ nanosheets; Figure S3: (a–c) HRTEM images of hcp-Ru/C_3_N_4_. (d–f) HRTEM images of fcc-Ru/C_3_N_4_; Figure S4: (a) Tauc plots and (b) band strctures of C_3_N_4_; Figure S5: (a) TEM image and (d) HRTEM image of hcp-Ru/C_3_N_4_ after reaction. (b) XRD patterns of hcp-Ru/C_3_N_4_ after reaction. (c) Size distribution histograms of Ru nanocrystals on the hcp-C_3_N_4_ nanosheets after reaction. (d) STEM and elemental mapping images of hcp-Ru/C_3_N_4_; Figure S6: (a) EIS Nyquist plots and (b) LSV curves of the catralysts. (c) Theoretical simulated charge difference distribution of hcp-Ru/C_3_N_4_ and fcc-Ru/C_3_N_4_; Figure S7: (a) Steady-state PL spectra and (b–d) N_2_ sorption isotherms of hcp-Ru/C_3_N_4_, fcc-Ru/C_3_N_4_ and C_3_N_4_; Table S1: The ICP-MS results of hcp-Ru/C_3_N_4_ and fcc-Ru/C_3_N_4_; Table S2: *EQE* Parameters and Corresponding Results; Table S3: Comparison of the photocatalytic activity and quantum efficiency over g-C_3_N_4_-based photocatalysts loaded with other materials. Refs. [32,45,46,47,48,49,50,51,52] can be found in Supplementary Materials.

## References

[1] Ben-Shahar Y., Scotognella F., Kriegel I., Moretti L., Cerullo G., Rabani E., Banin U. (2016). Optimal metal domain size for photocatalysis with hybrid semiconductor-metal nanorods. Nat. Commun..

[2] Ham R., Nielsen C.J., Pullen S., Reek J.N.H. (2023). Supramolecular Coordination Cages for Artificial Photosynthesis and Synthetic Photocatalysis. Chem. Rev..

[3] Zhou M., Li C., Fang J. (2021). Noble-Metal Based Random Alloy and Intermetallic Nanocrystals: Syntheses and Applications. Chem. Rev..

[4] Yang X., Zhang Y., Wang Y., Xin C., Zhang P., Liu D., Mamba B.B., Kefeni K.K., Kuvarega A.T., Gui J. (2020). Hollow β-Bi_2_O_3_@CeO_2_ heterostructure microsphere with controllable crystal phase for efficient photocatalysis. Chem. Eng. J..

[5] Wang Y., Yu W., Wang C., Chen F., Ma T., Huang H. (2024). Defects in photoreduction reactions: Fundamentals, classification, and catalytic energy conversion. eScience.

[6] Zhao M., Xia Y. (2020). Crystal-phase and surface-structure engineering of ruthenium nanocrystals. Nat. Rev. Mater..

[7] Yao Y., Wang S., Li Z., Wu Y. (2021). Atomic level engineering of noble metal nanocrystals for energy conversion catalysis. J. Energy Chem..

[8] Zhai W., Li Z., Wang Y., Zhai L., Yao Y., Li S., Wang L., Yang H., Chi B., Liang J. (2024). Phase Engineering of Nanomaterials: Transition Metal Dichalcogenides. Chem. Rev..

[9] Yi J., Zhu X., Zhou M., Zhang S., Li L., Song Y., Chen H., Chen Z., Li H., Xu H. (2020). Crystal phase dependent solar driven hydrogen evolution catalysis over cobalt diselenide. Chem. Eng. J..

[10] Chen Y., Lai Z., Zhang X., Fan Z., He Q., Tan C., Zhang H. (2020). Phase engineering of nanomaterials. Nat. Rev. Chem..

[11] Li H., Zhou X., Zhai W., Lu S., Liang J., He Z., Long H., Xiong T., Sun H., He Q. (2020). Phase Engineering of Nanomaterials for Clean Energy and Catalytic Applications. Adv. Energy Mater..

[12] Yun Q., Ge Y., Huang B., Wa Q., Zhang H. (2023). Ligand-Assisted Phase Engineering of Nanomaterials. ACC Chem. Res..

[13] Zhang Z., Liu G., Cui X., Chen B., Zhu Y., Gong Y., Saleem F., Xi S., Du Y., Borgna A. (2018). Crystal Phase and Architecture Engineering of Lotus-Thalamus-Shaped Pt-Ni Anisotropic Superstructures for Highly Efficient Electrochemical Hydrogen Evolution. Adv. Mater..

[14] Li P., Li W., Huang Y., Huang Q., Li J., Zhao S., Tian S. (2023). Unconventional Phase Synergies with Doping Engineering over Ni Electrocatalyst Featuring Regulated Electronic State for Accelerated Urea Oxidation. ChemSusChem.

[15] Yi J., Zhang G., Cao X., Zhu X., Li L., Wang X., Zhu X., Song Y., Xu H., Wang X. (2024). Structurally disordered MoSe_2_ with rich 1T phase as a universal platform for enhanced photocatalytic hydrogen production. J. Colloid Interface Sci..

[16] Pelicano C.M., Saruyama M., Takahata R., Sato R., Kitahama Y., Matsuzaki H., Yamada T., Hisatomi T., Domen K., Teranishi T. (2022). Bimetallic Synergy in Ultrafine Cocatalyst Alloy Nanoparticles for Efficient Photocatalytic Water Splitting. Adv. Funct. Mater..

[17] She P., Qin J.S., Sheng J., Qi Y., Rui H., Zhang W., Ge X., Lu G., Song X., Rao H. (2022). Dual-Functional Photocatalysis for Cooperative Hydrogen Evolution and Benzylamine Oxidation Coupling over Sandwiched-Like Pd@TiO_2_@ZnIn_2_S_4_ Nanobox. Small.

[18] Li R., Wu D., Rao P., Deng P., Li J., Luo J., Huang W., Chen Q., Kang Z., Shen Y. (2023). General approach for atomically dispersed precious metal catalysts toward hydrogen reaction. Carbon Energy.

[19] Zhang D., Zhang C., Ye B., Ma H., Wang C., Zhuang T., Lv Z. (2024). Droplet microreactor continuous synthesis of hierarchical Rh on CdZnS snowflakes for enhanced photocatalytic hydrogen evolution. Chem. Eng. J..

[20] Su K., Wang Y., Zhang C., Gao Z., Han J., Wang F. (2021). Tuning the Pt species on Nb_2_O_5_ by support-induced modification in the photocatalytic transfer hydrogenation of phenylacetylene. Appl. Catal. B Environ. Energy.

[21] Ding Z., Li X., Kang C., Yan S., Zhao D., Cai H., Zhang S.-Y., Zeng Y.-J. (2023). Single Ru atoms confined into MOF/C_3_N_4_ for dual improved photocatalytic carbon dioxide reduction and nitrogen fixation. Chem. Eng. J..

[22] Saito D., Yamazaki Y., Tamaki Y., Ishitani O. (2020). Photocatalysis of a Dinuclear Ru(II)-Re(I) Complex for CO_2_ Reduction on a Solid Surface. J. Am. Chem. Soc..

[23] Hamza M.A., Evans J.D., Andersson G.G., Metha G.F., Shearer C.J. (2024). Ultrathin Ru-CdIn_2_S_4_ nanosheets for simultaneous photocatalytic green hydrogen production and selective oxidation of furfuryl alcohol to furfural. Chem. Eng. J..

[24] Zhu S., Li Z., Hou L., Kim M.G., Jang H., Liu S., Liu X. (2023). Revealing The Role of Electronic Asymmetricity on Supported Ru Nanoclusters for Alkaline Hydrogen Evolution Reaction. Adv. Funct. Mater..

[25] Dong Y., Zhang W., Hu Z., Ng Y.H., Wei Z., Liu Y., Deng J., Dai H., Jing L. (2024). Advancing CO_2_ to CH_4_ conversion: The pivotal role of RuCu alloy in crystalline red phosphorus photocatalysis. Appl. Catal. B Environ. Energy.

[26] Nguyen Q.N., Kim E.M., Ding Y., Janssen A., Wang C., Li K.K., Kim J., Fichthorn K.A., Xia Y. (2024). Elucidating the Role of Reduction Kinetics in the Phase-Controlled Growth on Preformed Nanocrystal Seeds: A Case Study of Ru. J. Am. Chem. Soc..

[27] Lin J.-T., Liu Y.-H., Tsao C.-Y., Wu C.-Y., Hsieh C.-J., Chen M.-Z., Chang C.-W., Hsiao Y.-C., Chen H.-L., Yang T.-H. (2023). Toward a Quantitative Understanding of Crystal-Phase Engineering of Ru Nanocrystals. Chem. Mater..

[28] Fan Z., Guo X., Yang M., Jin Z. (2022). Mechanochemical preparation and application of graphdiyne coupled with CdSe nanoparticles for efficient photocatalytic hydrogen production. Chin. J. Catal..

[29] Yu B., Li H., White J., Donne S., Yi J.B., Xi S.B., Fu Y., Henkelman G., Yu H., Chen Z.L. (2020). Tuning the Catalytic Preference of Ruthenium Catalysts for Nitrogen Reduction by Atomic Dispersion. Adv. Funct. Mater..

[30] Liu D., Jiang L., Chen D., Hao Z., Deng B., Sun Y., Liu X., Jia B., Chen L., Liu H. (2024). Twin S-Scheme g-C_3_N_4_/CuFe_2_O_4_/ZnIn_2_S_4_ Heterojunction with a Self-Supporting Three-Phase System for Photocatalytic CO_2_ Reduction: Mechanism Insight and DFT Calculations. ACS Catal..

[31] Zhou G., Zhang L., Xia Y., Yin W., Zhu X., Hou J., Wang S., Ning X., Wang X. (2024). Remarkably enhanced hydrogen evolution of g-C_3_N_4_ nanosheet under simulated sunlight via AgPt alloy co-catalyst with low amount of Pt. J. Clean. Prod..

[32] Chen Z., Bu Y., Wang L., Wang X., Ao J.-P. (2020). Single-sites Rh-phosphide modified carbon nitride photocatalyst for boosting hydrogen evolution under visible light. Appl. Catal. B Environ. Energy.

[33] Kusada K., Kobayashi H., Yamamoto T., Matsumura S., Sumi N., Sato K., Nagaoka K., Kubota Y., Kitagawa H. (2013). Discovery of Face-Centered-Cubic Ruthenium Nanoparticles: Facile Size-Controlled Synthesis Using the Chemical Reduction Method. J. Am. Chem. Soc..

[34] Saruyama M., Pelicano C.M., Teranishi T. (2022). Bridging electrocatalyst and cocatalyst studies for solar hydrogen production via water splitting. Chem. Sci..

[35] Pelicano C.M., Tong H. (2023). Recent advances in cocatalyst engineering for solar-driven overall water splitting. Appl. Res..

[36] Zhang S., Yi J., Liu M., Shi L., Chen M., Wu L. (2024). High-Density Atomically Dispersed Metals Activate Adjacent Nitrogen/Carbon Sites for Efficient Ammonia Electrosynthesis from Nitrate. ACS Nano.

[37] Wang H., Xu C., Chen Q., Ming M., Wang Y., Sun T., Zhang Y., Gao D., Bi J., Fan G. (2019). Nitrogen-Doped Carbon-Stabilized Ru Nanoclusters as Excellent Catalysts for Hydrogen Production. ACS Sustain. Chem. Eng..

[38] Wang X., Li N., Wang G.C., Liu M., Zhang C., Liu S. (2024). Ultrafine Nanoclusters Unlocked 3d-4f Electronic Ladders for Efficient Electrocatalytic Water Oxidation. ACS Nano.

[39] Wu Y., Liu Y., Emrick T., Russell T.P. (2020). Polymer design to promote low work function surfaces in organic electronics. Prog. Polym. Sci..

[40] Hong S.J., Lee S., Jang J.S., Lee J.S. (2011). Heterojunction BiVO_4_/WO_3_ electrodes for enhanced photoactivity of water oxidation. Energy Environ. Sci..

[41] Guan Q., Ran W., Zhang D., Li W., Li N., Huang B., Yan T. (2024). Non-Metal Sulfur Doping of Indium Hydroxide Nanocube for Selectively Photocatalytic Reduction of CO_2_ to CH_4_: A “One Stone Three Birds” Strategy. Adv. Sci..

[42] Yi J., Fei T., Li L., Yu Q., Zhang S., Song Y., Lian J., Zhu X., Deng J., Xu H. (2021). Large-scale production of ultrathin carbon nitride-based photocatalysts for high-yield hydrogen evolution. Appl. Catal. B Environ. Energy.

[43] Zhou Z., Yang Y., Hu L., Zhou G., Xia Y., Hu Q., Yin W., Zhu X., Yi J., Wang X. (2022). Phase Control of Cobalt Selenide: Unraveling the Relationship Between Phase Property and Hydrogen Evolution Catalysis. Adv. Mater. Interfaces.

[44] Wang Y., Qian W., Zhou G., Zhang S., Zhu X., Li L., Zhu X., Wang X., Han X., Yi J. (2024). MOF-derived phase-selective synthesis of ln_2_O_3_ with appropriate surface atomic arrangement for CO_2_ photoreduction. Chem. Eng. J..

[45] Liu Y., Xu X., Li A., Si Z., Wu X., Ran R., Weng D. (2021). A strategy to construct (reduced graphene oxide, γ-Fe_2_O_3_)/C_3_N_4_ step-scheme photocatalyst for visible-light water splitting. Catal. Commun..

[46] Shen J., Luo C., Qiao S., Chen Y., Tang Y., Xu J., Fu K., Yuan D., Tang H., Zhang H. (2023). Single-Atom Cu Channel and N-Vacancy Engineering Enables Efficient Charge Separation and Transfer between C_3_N_4_ Interlayers for Boosting Photocatalytic Hydrogen Production. ACS Catal..

[47] Wang W., Bai X., Ci Q., Du L., Ren X., Phillips D.L. (2021). Near-Field Drives Long-Lived Shallow Trapping of Polymeric C_3_N_4_ for Efficient Photocatalytic Hydrogen Evolution. Adv. Funct. Mater..

[48] Xing F., Wang C., Liu S., Jin S., Jin H., Li J. (2023). Interfacial Chemical Bond Engineering in a Direct Z-Scheme g-C_3_N_4_/MoS_2_ Heterojunction. ACS Appl. Mater. Interfaces.

[49] Wang S., Chen L., Zhao X., Zhang J., Ao Z., Liu W., Wu H., Shi L., Yin Y., Xu X. (2020). Efficient photocatalytic overall water splitting on metal-free 1D SWCNT/2D ultrathin C_3_N_4_ heterojunctions via novel non-resonant plasmonic effect. Appl. Catal. B Environ. Energy.

[50] Shao M., Chen W., Ding S., Lo K.H., Zhong X., Yao L., Ip W.F., Xu B., Wang X., Pan H. (2019). WX_y_/g-C_3_N_4_ (WX_y_ = W_2_C, WS_2_, or W_2_N) Composites for Highly Efficient Photocatalytic Water Splitting. ChemSusChem.

[51] Tahir B., Tahir M., Alraeesi A., Kumar N., Al-Marzouqi M. (2024). Synergistic effect of bimetallic RuCo loaded N-defective g-C_3_N_4_ nanosheets with cleavage of metal-hydrogen bonds for H_2_ production in a continuous flow photoreactor. Int. J. Hydrogen Energy.

[52] Zhou X., Yu X., Peng L., Luo J., Ning X., Fan X., Zhou X., Zhou X. (2024). Pd(II) coordination molecule modified g-C_3_N_4_ for boosting photocatalytic hydrogen production. J. Colloid Interface Sci..

