# Supplementary material for: Phase-Controlled Synthesis of Ru Supported on Carbon Nitride and the Application in Photocatalytic H2 Evolution
Source: Materials (Basel). 2025 Mar 13;18(6):1259. doi: 10.3390/ma18061259 (PMC11943884; doi:10.3390/ma18061259)
Supplement: Supplementary file 1 [file materials-18-01259-s001.zip › materials-3514690-supplementary.pdf]

Supplementary Materials

Phase-controlled synthesis of Ru supported on carbon nitride and the application in photocatalytic H_2 evolution

Xiaohu Sun [1], Xiangyang Cao [1], Ganghua Zhou [1], Tiaolong Lv [1], Jian Xu [1], Yubo Zhou [2], Zhigang Wang [1,*] and Jianjian Yi [1,2,3,*]

[1] College of Environmental Science and Engineering, Yangzhou University, Yangzhou 225127, China
[2] Ningbo Solartron Technology Co., Ltd., Ningbo 315034, China
[3] School of Material Science and Engineering, Jiangsu University, Zhenjiang 212013, China
* Correspondence: wangzg@yzu.edu.cn (Z.W.); jjyi@yzu.edu.cn (J.Y.)

Characterization

The crystal phase was performed by X-ray diffraction (XRD) on a Bruker D8 Advanced X-ray Diffractometer (Cu-Kα radiation: λ = 0.15406 nm). High-resolution transmission electron microscope (HRTEM) images and elemental mapping were captured on a Tecnai G2 F30 S-TWIN at an acceleration voltage of 300 kV. Atomic force microscopy (AFM) images were obtained by SPM-9700HT. X-ray photoelectron spectroscopy (XPS) were carried out on a Thermo Scientific ESCALAB 250 apparatus. The light absorption capacity was tested by Cary 5000 UV-visible-near-infrared absorption spectrometer. Photoluminescence (PL) spectra were acquired using an Edinburgh FLS1000 fluorescence spectrometer. The micromorphology of the sample was observed by field emission scanning electron microscope (FE-SEM, S-4800II) under the acceleration voltage of 5.0 kV. The specific surface area was measured via the Brunauer-Emmett-Teller (BET) method by N_2 adsorption–desorption isotherms using a Micromeritics ASAP 2460 instrument. Inductively coupled plasma mass spectrometry (ICP-MS, Elan DRC-e) was utilized to determine the Ru content on the C_3N_4 nanosheets.

Photo-electrochemical test

The photocurrent test used the Donghua DH7000 electrochemical testing system, which is equipped with a standard three-electrode system. Initially, 20 mg catalysts were added to a mixture of 1 mL naphthol, ethanol, and DI water (volume ratio 1:5:14), dispersed uniformly by sonication. Then, 20 μL of the mixture was taken with a pipette and sprinkled on an indium tin oxide (ITO) glass, which had an area of 1 cm × 1 cm. The ITO glass loaded with the catalyst was first dried under an infrared lamp and then heated in an oven at 80°C for 1 h. The photocurrent response and impedance spectroscopy of the catalyst were tested on an electrochemical workstation, with a 300 W xenon lamp as the light source, Ag/AgCl electrode as the reference electrode, Pt wire electrode as the counter electrode, and the ITO glass loaded with the catalyst as the working electrode. The PEC of as-prepared catalysts was evaluated by electrochemical impedance spectroscopy (EIS), transient photocurrent test (I-t). The applied bias during the test was -0.2 V, and the electrolyte was a 0.1M Na_2SO_4 solution. Mott–Schottky (M–S) plots were also measured using the same three-electrode system, over an alternating current frequency range of 500, 1000, and 1500 Hz, in a 0.1 M Na_2SO_4 aqueous solution.

Theoretical simulation methods

The density functional theory (DFT) calculations were carried out using the Vienna ab initio simulation package (VASP). The exchange–correlation energy was described using the Perdew–Burke–Ernzerhof (PBE). The PBE functional combined with Projected Augmented Wave (PAW) pseudopotentials was used. A 400 eV plane-wave kinetic energy cut off was selected, and a 3 × 3 × 1 Monhorst-Pack *k* point sampling was adopted for the structure relaxation. The p(3×3) supercell model was constrcuted, and the thickness of the vacuum layer along the c-axis was 15 Å. A residual force threshold of 0.05 eV $Å^{-1}$ was set for geometry optimizations. The calculated surfaces are in (101) and (111) in the model building process.

The Gibbs free-energy (ΔG_{H^*}) is expressed as: $\Delta G_{H^*} = \Delta E_{H^*} + \Delta E_{ZPE} - T\Delta S$. Where ΔE_{H^*}, ΔE_{ZPE} and ΔS are the adsorption energy of atomic hydrogen on the given surface, zero point energy correction and entropy change of H* adsorption, respectively. The zero point energy correction can be estimated by the equation $\Delta E_{ZPE} = E_{ZPE}(H^*) - 1/2E_{ZPE}(H_2)$. ΔS can be calculated by the equation $\Delta S = S(H^*) - 1/2S(H_2) \approx -1/2S(H_2)$, due to the negligible entropy of hydrogen in its adsorbed state. ΔE_{H^*} is calculated as $\Delta E_{H^*} = E_{tot} - E_{sub} - 1/2E_{H_2}$, where E_{tot} and E_{sub} are energies of H absorbed system and the clean given surface, and E_{H_2} is the energy of H_2 molecular in gas phase.

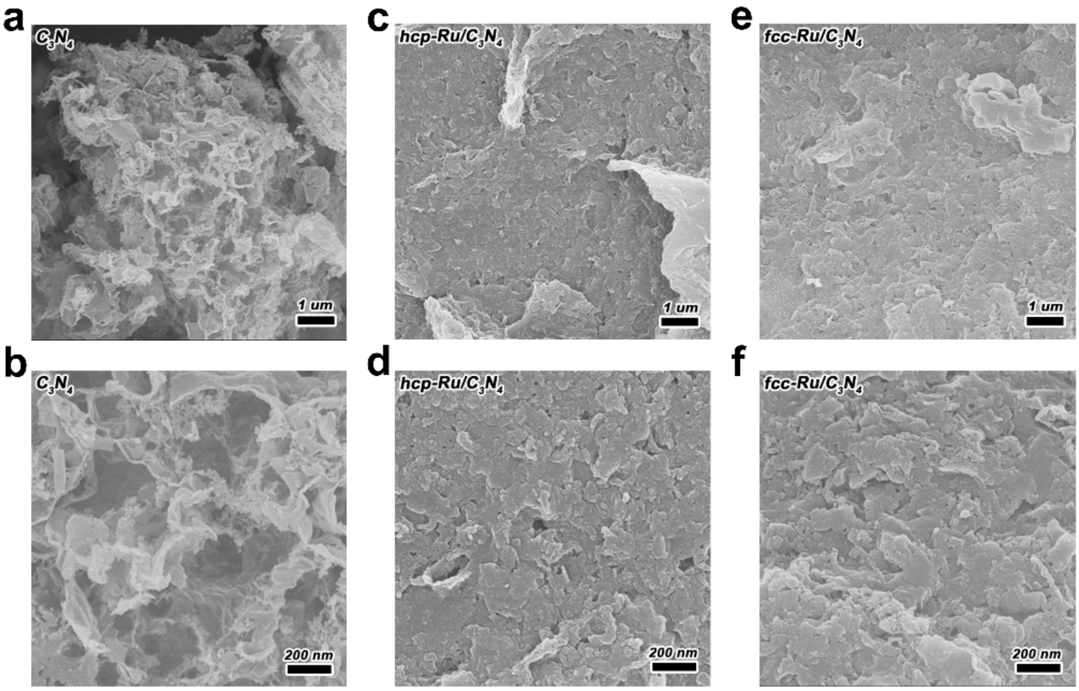

Figure S1. SEM images of C_3N_4, hcp-Ru/C_3N_4 and fcc-Ru/C_3N_4.

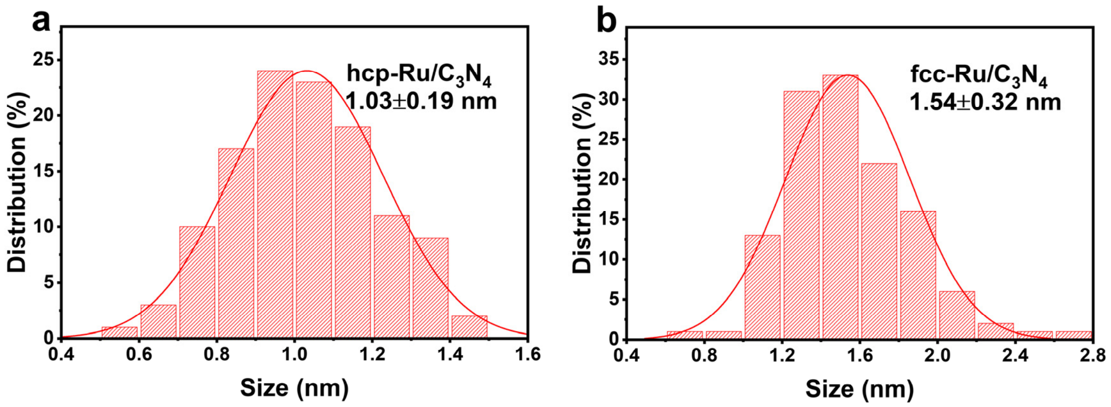

Figure S2. (**a, b**) Size distribution histograms of Ru nanocrystals on the C_3N_4 nanosheets.

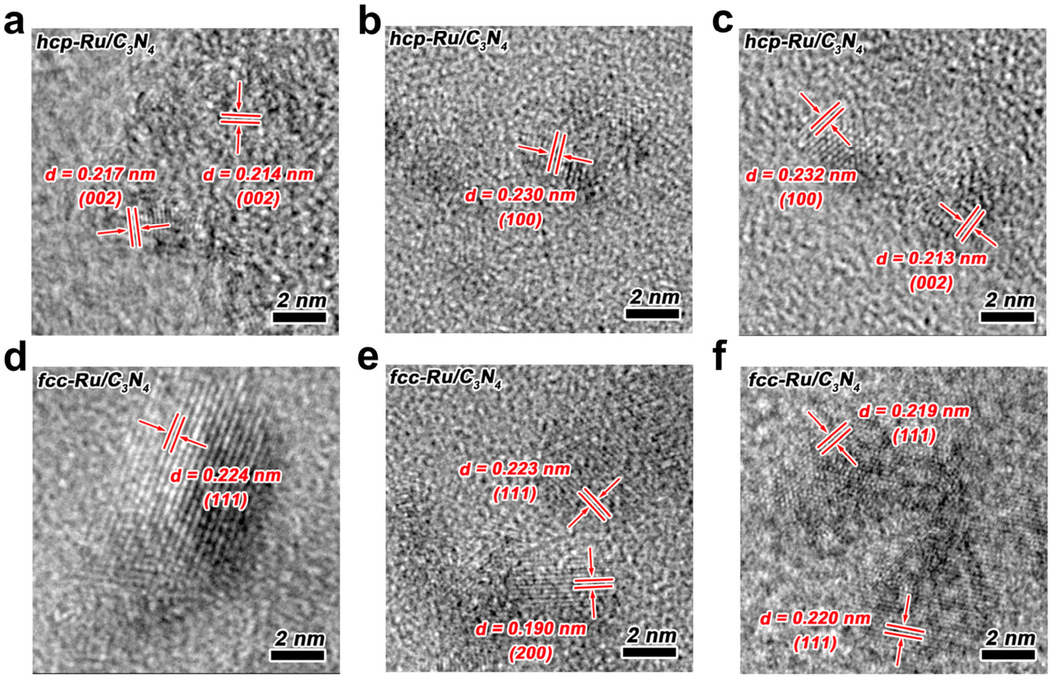

Figure S3. (**a-c**) HRTEM images of hcp-Ru/C_3N_4. (**d-f**) HRTEM images of fcc-Ru/C_3N_4.

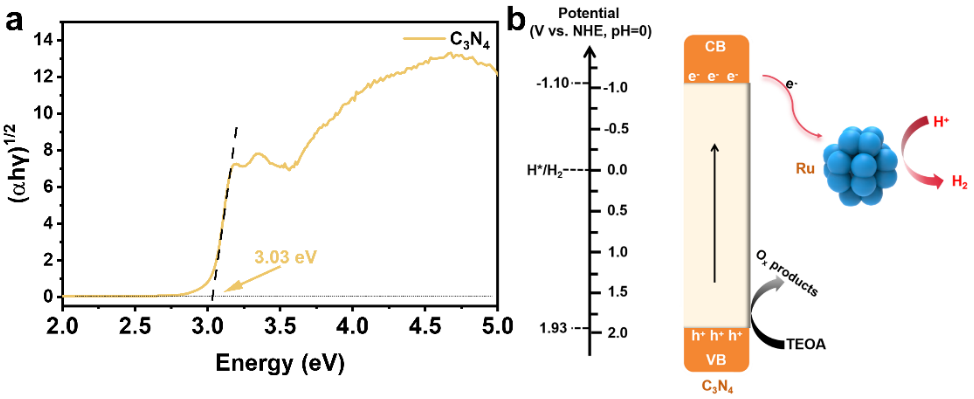

Figure S4. (**a**) Tauc plots and (**b**) band strctures of C_3N_4.

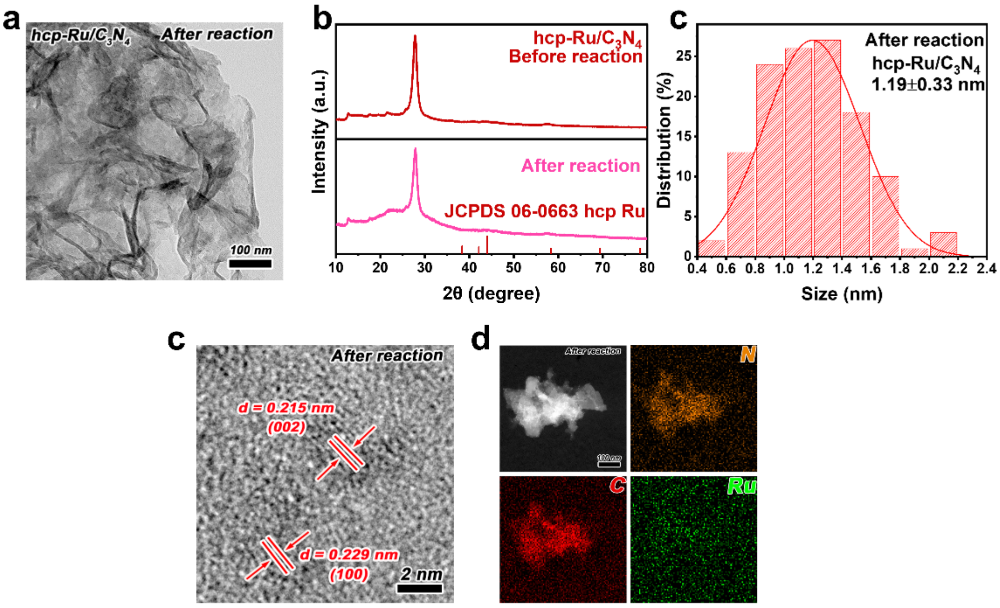

Figure S5. (**a**) TEM image and (**d**) HRTEM image of hcp-Ru/C_3N_4 after reaction. (**b**) XRD patterns of hcp-Ru/C_3N_4 after reaction. (**c**). Size distribution histograms of Ru nanocrystals on the hcp-C_3N_4 nanosheets after reaction. (**d**) STEM and elemental mapping images of hcp-Ru/C_3N_4.

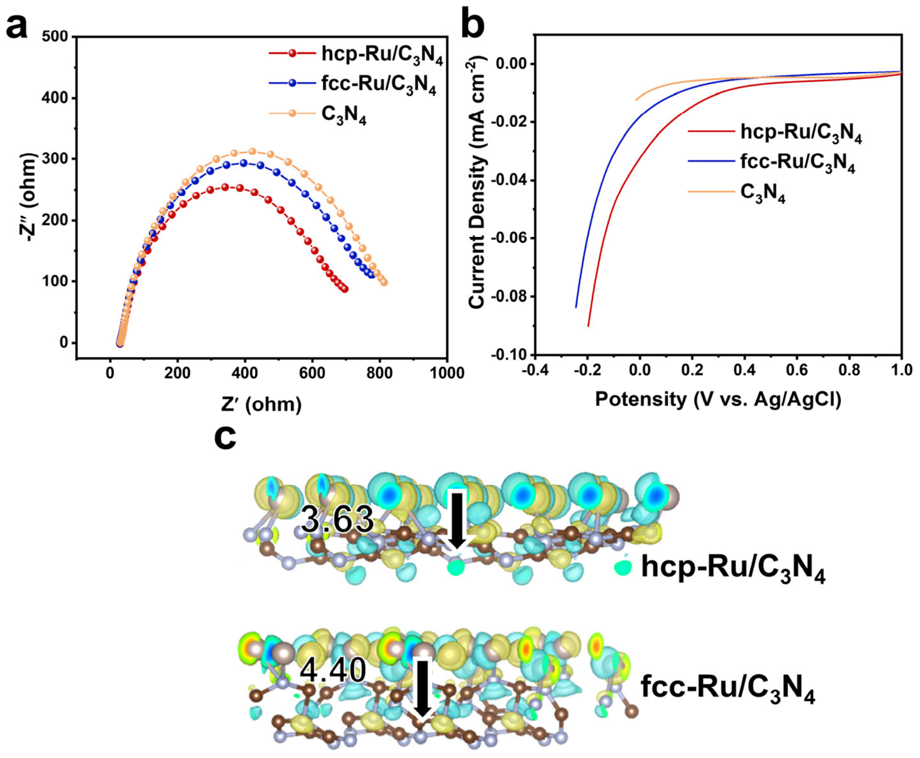

Figure S6. (**a**) EIS Nyquist plots and (**b**) LSV curves of the catralysts. (**c**) Theoretical simulated charge difference distribution of hcp-Ru/C_3N_4 and fcc-Ru/C_3N_4.

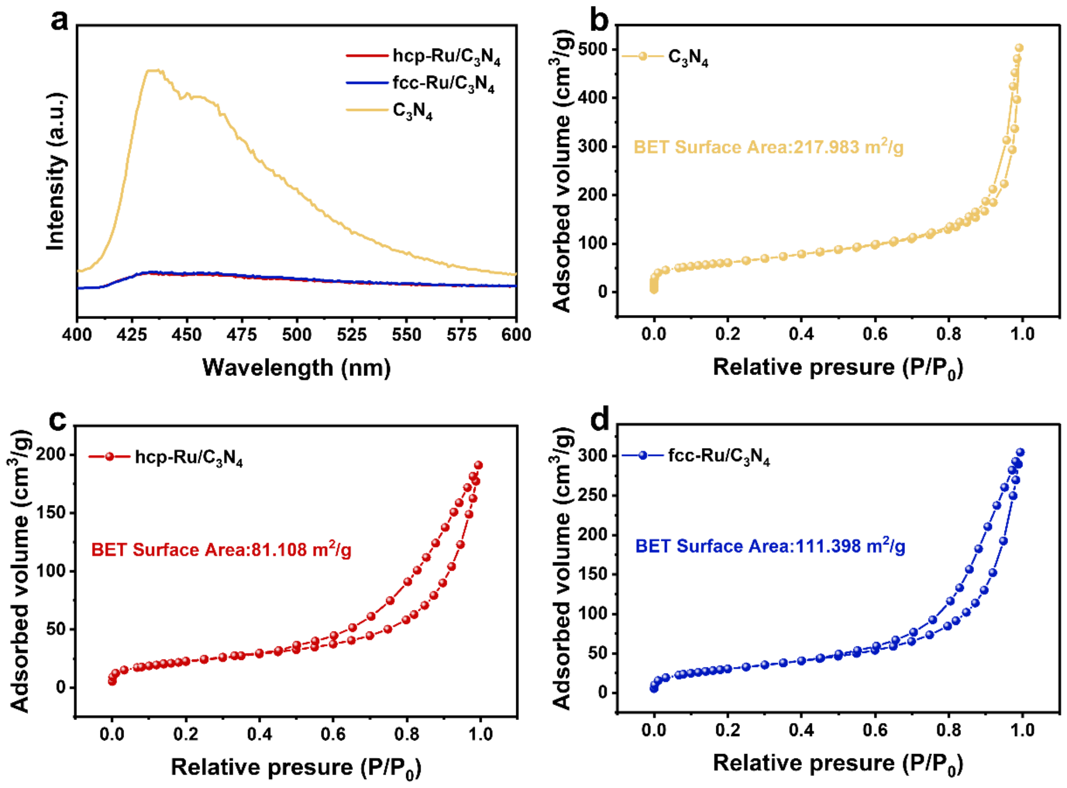

Figure S7. (**a**) Steady-state PL spectra and (**b-d**) N_2 sorption isotherms of hcp-Ru/C_3N_4, fcc-Ru/C_3N_4 and C_3N_4.

Table S1. The ICP-MS results of hcp-Ru/C_3N_4 and fcc-Ru/C_3N_4.

Content / Catalysts	Ru (wt%)
hcp-Ru/C_3N_4	3.53
fcc-Ru/C_3N_4	3.64

Calculation of external quantum efficiency

The external quantum efficiency (*EQE*) was measured by inserting a band pass filter of 420 nm in front of a light source. And the other conditions are similar to the one for hydrogen evolution measurement.

The radius of light spot is 4.5 cm

The area of light spot is 63.59 cm^2

The light intensity is determined as follows:

$$E^* = \frac{P}{2.06 \times S}$$

Where E^*, P, 2.06 and S denote the light intensity, radiation power, instrument attenuation coefficient and light-receiving area of the detector.

The radiation power can be measured using a optical power meter (PL-MW2000, Beijing Perfectlight Technology Co., Ltd.).

The external quantum efficiency (*EQE*) values were determined using the follow equation:

$$EQE = \frac{the\ number\ of\ reacted\ electrons}{the\ number\ of\ incident\ photons} \times 100\%$$

$$= \frac{2 \times the\ number\ of\ evolved\ H_2\ molecules}{N} \times 100\%$$

Total number of reacted electrons = 2×the number of evolved H_2 molecules

The numbers of photons were counted according to the follow equation:

$$N = \frac{E\lambda}{hc}$$

Where E, λ, h and c denote the total light intensity, wavelength, Planck constant and velocity of light.

The total light intensity = E^*×S

Hydrogen evolution per unit area = detected hydrogen molecules (μmol)/S

Reaction time: 1 h

Table S2. *EQE* Parameters and Corresponding Results.

**Wavelength**	**H_2 Evolved (μmol/cm²/h)**	**Light Intensity (mW/cm²)**	*EQE*
λ = 420 nm	1.573	4.718	5.28%

Table S3. Comparison of the photocatalytic activity and quantum efficiency over g-C_3N_4-based photocatalysts loaded with other materials.

Photocatalyst	Light Source	Reaction Conditions	H_2 Evolution Rate (μmol g^{-1} h^{-1})	Quantum Efficiency	Reference
hcp-Ru/C_3N_4	300 W Xe lamp	50 mL of 10 *vol.* % TEOA aqueous solution	2422.84	5.28% (420 nm)	This work
(rGO, γ-Fe_2O_3)/ C_3N_4	280 W Xe lamp with an optical filter (λ > 420 nm)	0.1 M $AgNO_3$ solution	23.30	15.2% (420 nm)	[1]
Rh-P/CN (H)	300 W Xe lamp with a 400 nm cut-off filter	100 mL of 10 *vol.* % TEOA aqueous solution	2078.50	N/A	[2]
SA-Cu-CN-620	300 W Xe lamp with a 420 nm cut-off filter	80 mL of 10 *vol.* % TEOA aqueous solution	605.14	31.6% (420 nm)	[3]
Pt/In_2O_3-cube/g-C_3N_4	300 W Xe lamp ($\lambda \geq$ 400 nm)	50 mL of 10 *vol.* % TEOA aqueous solution	1917.00	7.73% (420 nm)	[4]
MCN-20 (g-C_3N_4/MoS_2 (20 mg))	300 W Xe lamp (AM 1.5 filter, light intensity 450 mW/cm^2)	100 mL of 20 *vol.* % TEOA aqueous solution	590	N/A	[5]
1D SWCNT/2D C_3N_4	300 W Xe lamp (λ > 420 nm)	25 mL of 10 *vol.* % TEOA aqueous solution; 3.0 wt% Pt	1346	5.20% (420 nm)	[6]
W_2C/g-C_3N_4	solar simulator (AM 1.5 filter, light intensity 100 mW/cm^2)	85 mL of 10 *vol.* % TEOA aqueous solution	1960	1.52% (420 nm)	[7]
Ru–Co/N–C_3N_4	35 W HID lamp	100 mL of 5 *vol.* % methanol solution	4450	N/A	[8]
R-TAP-Pd(II)@g-C_3N_4	300 W Xe lamp with a 420 nm cut-off filter	100 mL of 15 *vol.* % methanol solution	1085	4.85% (420 nm)	[9]

References

1. Liu, Y.; Xu, X.; Li, A.; Si, Z.; Wu, X.; Ran, R., Weng, D. A strategy to construct (reduced graphene oxide, γ-Fe_2O_3)/C_3N_4 step-scheme photocatalyst for visible-light water splitting. *Catal. Commun.* **2021**, 157, 106327.
2. Chen, Z.; Bu, Y.; Wang, L.; Wang, X., Ao, J.-P. Single-sites Rh-phosphide modified carbon nitride photocatalyst for boosting hydrogen evolution under visible light. *Appl. Catal. B Environ. Energy*. **2020**, 274, 119117.
3. Shen, J.; Luo, C.; Qiao, S.; Chen, Y.; Tang, Y.; Xu, J.; Fu, K.; Yuan, D.; Tang, H.; Zhang, H., Liu, C. Single-Atom Cu Channel and N-Vacancy Engineering Enables Efficient Charge Separation and Transfer between C_3N_4 Interlayers for Boosting Photocatalytic Hydrogen Production. *ACS Catal.* **2023**, 13, 6280-8.
4. Wang, W.; Bai, X.; Ci, Q.; Du, L.; Ren, X., Phillips, D.L. Near-Field Drives Long-Lived Shallow Trapping of Polymeric C_3N_4 for Efficient Photocatalytic Hydrogen Evolution. *Adv. Funct. Mater.* **2021**, 31, 2103978.
5. Xing, F.; Wang, C.; Liu, S.; Jin, S.; Jin, H., Li, J. Interfacial Chemical Bond Engineering in a Direct Z-Scheme g-C_3N_4/MoS_2 Heterojunction. *ACS Appl. Mater. Interfaces*. **2023**, 15, 11731-40.
6. Wang, S.; Chen, L.; Zhao, X.; Zhang, J.; Ao, Z.; Liu, W.; Wu, H.; Shi, L.; Yin, Y.; Xu, X.; Zhao, C.; Duan, X.; Wang, S., Sun, H. Efficient photocatalytic overall water splitting on metal-free 1D SWCNT/2D ultrathin C_3N_4 heterojunctions via novel non-resonant plasmonic effect. *Appl. Catal. B Environ. Energy*. **2020**, 278, 119312.
7. Shao, M.; Chen, W.; Ding, S.; Lo, K.H.; Zhong, X.; Yao, L.; Ip, W.F.; Xu, B.; Wang, X., Pan, H. WX_y/g-C_3N_4 (WX_y = W_2C, WS_2 , or W_2N) Composites for Highly Efficient Photocatalytic Water Splitting. *ChemSusChem*. **2019**, 12, 3355-62.
8. Tahir, B.; Tahir, M.; Alraeesi, A.; Kumar, N., Al-Marzouqi, M. Synergistic effect of bimetallic RuCo loaded N-defective g-C_3N_4 nanosheets with cleavage of metal-hydrogen bonds for H_2 production in a continuous flow photoreactor. *International Journal of Hydrogen Energy*. **2024**, 95, 402-16.
9. Zhou, X.; Yu, X.; Peng, L.; Luo, J.; Ning, X.; Fan, X.; Zhou, X., Zhou, X. Pd(II) coordination molecule modified g-C_3N_4 for boosting photocatalytic hydrogen production. *J. Colloid Interface Sci.* **2024**, 671, 134-44.
